# Book Reviews

**Published:** 1894-05

**Authors:** 


### *BOOK REVIEWS.*

A Primer of Psychology and Mental Disease.—By C. B. Burr, M.D., Medical Superintendent of the Eastern Michigan Asylum. Price $1.00. George S. Davis, Publisher, Detroit, Mich.

This primer has been prepared to supply the need of an elementary work on psychology and mental disease for the class-room. It discusses clearly and concisely the fundamental principles of mental physiology and also the most common forms of mental disease with their causes and treatment. Part III. deals in a very practical manner with the management of insane patients.

---

The Funny Bone.—A book of mirth for doctors, medical students, druggists, etc. Edited by Dr. L. Crusius, St. Louis. Price, fifty cents. Funny Bone Publishing Co., St. Louis, 1421 Market street.

There is fully a half dollar's worth of fun in this funny book. The jokes are generally good, and for the most part new, although some have been drifting around among the journals for some months. It is well supplied with original illustrations by the editor. The busy doctor who can appreciate some medicated humor will do well to lay down his journals and books sometimes and read the "Funny Bone."

---

The Discovery of Modern Anesthesia.—By Laird W. Nevius, M.D., New York. Published by Laird W. Nevius, M.D., Cooper Institute, New York. Price, $1.00.

The history of this great discovery has been the subject of much dispute, and the publication of such a work as this, setting forth the main facts in the case of each of the claimants, is very much in order. The author does not seek to advocate the claim of either of the gentlemen whose names have been associated with this discovery. He has written from a desire to be wholly impartial, his handling of history is correct, so far as we are acquainted with it, and the reader is left to judge upon whom rests the honor of priority. The friends of Dr. Crawford W. Long will be pleased to know that the evidence as here presented is in his favor. This little book will serve a good purpose in aiding to clear up some of the confused ideas which exist relative to the discovery of anesthesia.

